# Undiagnosed Cirrhosis and Hepatic Encephalopathy in a National Cohort of Veterans With Dementia

**DOI:** 10.1001/jamanetworkopen.2023.53965

**Published:** 2024-01-31

**Authors:** Jasmohan S. Bajaj, Scott G. Silvey, Shari Rogal, Jacqueline G. O’Leary, Heather Patton, Timothy R. Morgan, Gowthami Kanagalingam, Angela Gentili, Michael Godschalk, Nilang Patel

**Affiliations:** 1 Department of Medicine, Virginia Commonwealth University, Richmond; 2 Richmond VA Medical Center, Richmond, Virginia; 3 Department of Biostatistics, Virginia Commonwealth University, Richmond; 4 Department of Medicine, University of Pittsburgh, Pittsburgh, Pennsylvania; 5 Pittsburgh VA Medical Center, Pittsburgh, Pennsylvania; 6 Department of Medicine, Dallas VA Medical Center, Dallas, Texas; 7 Department of Medicine, San Diego VA Medical Center, San Diego, California; 8 Medical Service, VA Long Beach Healthcare System, Long Beach, California; 9 Division of Geriatrics, Virginia Commonwealth University, Richmond

## Abstract

**Importance:**

Dementia and hepatic encephalopathy (HE) are challenging to distinguish clinically. Undiagnosed cirrhosis in a patient with dementia can lead to missed opportunities to treat HE.

**Objective:**

To examine the prevalence and risk factors of undiagnosed cirrhosis and therefore possible HE in veterans with dementia.

**Design, Setting, and Participants:**

A retrospective cohort study was conducted between 2009 and 2019 using data from the Veterans Health Administration (VHA) and 2 separate validation cohorts from the Richmond Veterans Affairs Medical Center. Data analysis was conducted from May 20 to October 15, 2023. Participants included 177 422 US veterans with a diagnosis of dementia at 2 or more clinic visits, no prior diagnosis of cirrhosis, and with sufficient laboratory test results to calculate the Fibrosis-4 (FIB-4) score.

**Exposures:**

Demographic and clinical characteristics.

**Main Outcomes and Measures:**

An FIB-4 score (>2.67 suggestive of advanced fibrosis and >3.25 suggestive of cirrhosis), capped at age 65 years even for those above this cutoff who were included in the analysis.

**Results:**

Among 177 422 veterans (97.1% men; 80.7% White; mean (SD) age, 78.35 [10.97] years) 5.3% (n = 9373) had an FIB-4 score greater than 3.25 and 10.3% (n = 18 390) had an FIB-4 score greater than 2.67. In multivariable logistic regression models, FIB-4 greater than 3.25 was associated with older age (odds ratio [OR], 1.07; 95% CI, 1.06-1.09), male gender (OR, 1.43; 95% CI, 1.26-1.61), congestive heart failure (OR, 1.48; 95% CI, 1.43-1.54), viral hepatitis (OR, 1.79; 95% CI, 1.66-1.91), Alcohol Use Disorders Identification Test score (OR, 1.56; 95% CI, 1.44-1.68), and chronic kidney disease (OR, 1.11; 95% CI, 1.04-1.17), and inversely associated with White race (OR, 0.79; 95% CI, 0.73-0.85), diabetes (OR, 0.78; 95% CI, 0.73-0.84), hyperlipidemia (OR, 0.84; 95% CI, 0.79-0.89), stroke (OR, 0.85; 95% CI, 0.79-0.91), tobacco use disorder (OR, 0.78; 95% CI, 0.70-0.87), and rural residence (OR, 0.92; 95% CI, 0.87-0.97). Similar findings were associated with the FIB-4 greater than 2.67 threshold. These codes were associated with cirrhosis on local validation. A local validation cohort of patients with dementia showed a similar percentage of high FIB-4 scores (4.4%-11.2%).

**Conclusions and Relevance:**

The findings of this cohort study suggest that clinicians encountering patients with dementia should be encouraged to screen for cirrhosis using the FIB-4 score to uncover reversible factors associated with cognitive impairment, such as HE, to enhance outcomes.

## Key Points

**Question:**

Could patients with dementia have undiagnosed cirrhosis and possibly missed treatable cognitive impairment?

**Findings:**

In this cohort study including 177 422 veterans with a diagnosis of dementia, a high Fibrosis-4 score suggestive of advanced liver fibrosis to cirrhosis was observed in 5% to 10% of the patients who previously did not have a diagnosis of cirrhosis. These findings were supported by 2 validation cohorts within the Richmond VA Medical Center.

**Meaning:**

The findings of this study suggest that clinicians treating patients with dementia should investigate a reversible factor associated with cognitive decline, including missed or undiagnosed cirrhosis with hepatic encephalopathy.

## Teaser

This cohort study examines the presence of undiagnosed cirrhosis and hepatic encephalopathy in US veterans with dementia.

## Introduction

Dementia poses a major clinical, psychosocial, and financial burden worldwide,^1,2^ with a suboptimal therapeutic landscape. Metabolic encephalopathies, such as hepatic encephalopathy (HE), are reversible with therapy, although their diagnosis requires a high index of suspicion.^3^ Major changes in the epidemiologic factors of liver disease are attributed to widespread advances for viral hepatitis care with increasing rates of metabolic syndrome and alcohol use disorder (AUD).^4^ However, a substantial proportion of advanced liver disease remains undiagnosed, especially among US veterans, who have major risk factors for both cirrhosis and dementia.^5,6^

Many veterans receiving Veterans Administration care are aging and often have comorbid conditions that affect cognitive function, such as AUD, posttraumatic stress disorder (PTSD), and head injuries, in addition to risk factors for cirrhosis.^7,8^ Among veterans with cirrhosis, concomitant dementia is common and is challenging to distinguish from HE, but the extent to which patients with diagnoses of dementia also have undiagnosed cirrhosis and HE is unknown.^9^ Undiagnosed cirrhosis among veterans with dementia could raise the possibility that part of their cognitive impairment may be due to reversible HE.^10^ Therefore, the determinants of undiagnosed cirrhosis among veterans with dementia is important to identify those eligible for screening and subsequent HE therapy.

Our aims were to examine in a national cohort of US veterans with dementia without previously diagnosed cirrhosis the prevalence of Fibrosis-4 (FIB-4) score greater than 3.25 suggestive of undiagnosed cirrhosis and FIB-4 greater than 2.67 suggestive of advanced liver fibrosis, identify characteristics related to this diagnosis, and validate these findings in 2 cohorts from the Richmond Veterans Affairs Medical Center.

## Methods

### Data Collection

Using the VHA Corporate Data Warehouse (CDW), we extracted information on outpatient or inpatient diagnoses of dementia from 2009 to 2019. We limited our study analysis up to 2019 to minimize the confounding effect of the COVID-19 pandemic. This cohort study followed the Strengthening the Reporting of Observational Studies in Epidemiology (STROBE) reporting guideline and was approved by the Richmond VAMC IRB. A waiver of informed consent was granted because this was deidentified database research.

We used validated *International Classification of Diseases, 9th Revision* (*ICD-9*) and *International Statistical Classification of Diseases and Related Health Problems, 10th Revision* (*ICD-10*) codes to identify patients with 2 or more dementia diagnoses at different dates.^9^ We then collected the index date, considered to be the second date of diagnosis, and removed patients with evidence of an outpatient or inpatient cirrhosis diagnosis or complication from cirrhosis using validated *ICD-9* and *ICD-10* codes, and/or lactulose or rifaximin prescriptions during the study period (eAppendix in Supplement 1).^11^ Patients without alanine aminotransferase (ALT) or aspartate aminotransferase (AST) levels or values over 250 U/L (to convert to microkatals per liter, multiply by 0.0167), as well as patients without platelet counts or with abnormally low platelet counts (≤50 × 10^9^/μL; to convert to 10^9^ per liter, multiply by 1), were excluded.

We calculated the FIB-4 score for each patient using the most recent AST, ALT, and platelet values that were closest to the index date during the 2 years after the index dementia date. Age is in the numerator of the FIB-4 score calculation; hence, higher age could lead to an erroneously high FIB-4 score.^12^ Due to this limitation of the FIB-4 score for any patients older than 65 years, we entered 65 years as an input variable (rather than their actual age) to calculate the FIB-4 score. Therefore, we did not exclude any patient solely due to age. We collected demographic data, including age, US region (reference level, North Atlantic) of VHA, residence status (urban vs rural), sex, body mass index, race and ethnicity, and comorbidities (details and codes the eAppendix in Supplement 1). Along with other parameters, data on race and ethnicity could be helpful in focusing which patient groups are likely to have a higher proportion of undiagnosed cirrhosis. Alcohol Use Disorders Identification Test (AUDIT-C) scores were obtained, and problem drinking was defined by a score of greater than 4 in men or greater than 3 in women per VHA guidance.^13^ All comorbidities were collected up to 2 years before the index diagnosis date of dementia; FIB-4 scores greater than 3.25 and greater than 2.67 were calculated, and cohorts above or below these cutoffs were compared.

For validation, we extracted data on Richmond VAMC patients included in this cohort to determine the reasons for high FIB-4 scores. Two clinicians (J.S.B. and G.K.) reviewed each medical record in detail. A second cohort of patients with dementia seen by the geriatrics clinic at the Richmond VAMC between January 1 and June 30, 2023, who were still alive underwent a detailed medical records review to examine the presence of high FIB-4 scores and potential causes.

### Statistical Analysis

Data analysis was performed from May 20 to October 15, 2023. Continuous variables are expressed as the mean (SD). The Charlson Comorbidity Index (CCI) score is expressed as the median (IQR), due to both the discrete nature and skewed distribution. Categorical variables are expressed as counts and percentages of the total. We used 2-sample *t* tests, Pearson χ^2^ tests, or the Wilcoxon rank sum test as appropriate to compare characteristics between the groups.

Statistical significance was defined as *P* < .05 for all hypothesis tests conducted, and all tests were 2-sided. RStudio, version 4.1.2 (R Foundation for Statistical Computing) was used for all statistical analysis.

All characteristics that were significantly different between the high FIB-4 and low FIB-4 groups (≥/<3.25 and≥/<2.67) in univariable models were included in multivariable logistic regression models. Age was capped at 65 years within these analyses, although individuals older than 65 years were included.

## Results

### CDW Cohort Description

We identified 287 257 veterans with dementia, of whom 38% were ineligible, resulting in 177 422 patients in the cohort (Figure). The major reasons for exclusion were lack of laboratory test values (AST, ALT, and platelet counts; n = 75 368) for FIB-4 calculation, diagnosed cirrhosis (n = 33 251), and abnormal laboratory test values (platelet count ≤50 × 10^3^/μL [n = 383], AST≥250 U/L [n = 403], and ALT≥250 U/L [n = 430]). Their mean (SD) age was 78.35 (10.97) years, 97.1% were male, 2.9% were female, 80.7% were White, 5.3% (n = 9373) had an FIB-4 score greater than 3.25, and 10.3% (n = 18 390) had an FIB-4 score greater than 2.67. Patients with an FIB-4 score greater than 3.25 were more likely to be older, male, Hispanic, and reside in the Southeast; patients with FIB-4 greater than 3.25 were less likely to be White, live in rural areas, and less likely to be from the Midwest (Table 1). Patients with a high FIB-4 score had a higher CCI score for comorbid conditions, the most common of which were chronic kidney disease (CKD) and cardiovascular conditions. Veterans with FIB-4 scores greater than 3.25 also had lower percentages of tobacco use disorder, sleep apnea, PTSD, head injury, and major depressive disorder. While lower in proportion than these comorbid conditions, patients with high FIB-4 scores had higher AUDIT-C scores, rates of prior hepatitis B and C, and HIV infection. Homelessness was not different between groups, while patients with high FIB-4 scores had a significantly lower mean body mass index than the rest. Similar results were seen using the greater than FIB-4 2.67 cutoff (Table 2).

**Figure.**
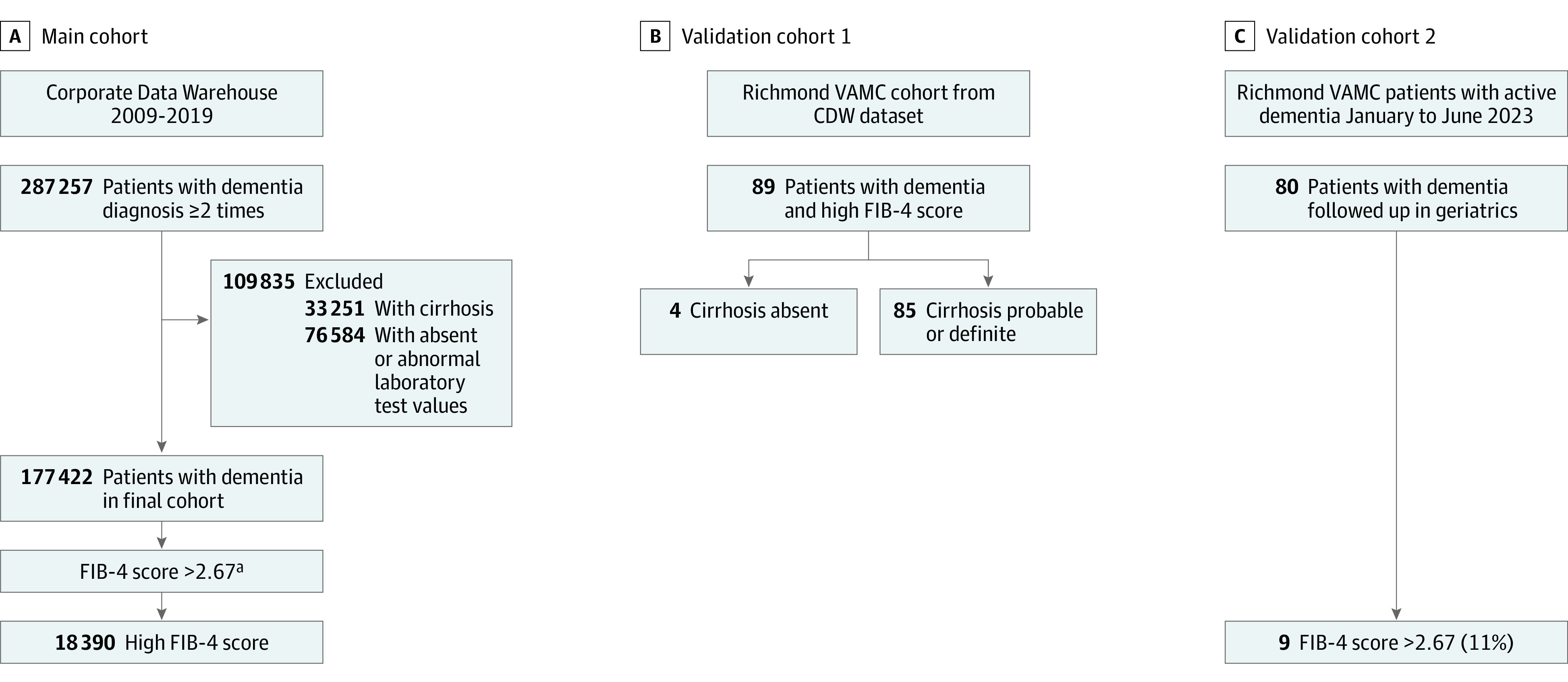
Flowchart of Patients With Dementia Diagnosis and Fibrosis-4 (FIB-4) Scores Across the Corporate Data Warehouse (CDW) and 2 Validation Cohorts ^a^Using FIB-4 >3.25 threshold, 9373 (5.3%) had high FIB-4.

**Table 1.** Characteristics of Patients With FIB-4 Level Greater Than 3.25 vs Patients With FIB-4 Level 3.25 or Less

Variable	No. (%)		*P* value
	FIB-4 > 3.25	FIB-4 ≤ 3.25	
Patients	9373 (5.3)	168 049 (94.7)	NA
Age, mean (SD), y	80.78 (9.65)	78.21 (11.02)	<.001
Sex			
Male	9211 (98.3)	163 148 (97.1)	<.001
Female	162 (1.7)	4901 (2.9)	
Race (White)	6726 (75.2)	129 266 (80.4)	<.001
Ethnicity (Hispanic or Latino)	654 (7.3)	10 872 (6.7)	.04
BMI, mean (SD)	25.78 (5.28)	26.73 (5.40)	<.001
Charlson Comorbidity Index, median (IQR)	3.00 (2.00-5.00)	3.00 (2.00-5.00)	<.001
Diabetes	3471 (37.0)	65 296 (38.9)	<.001
Hypertension	7511 (80.1)	128 989 (76.8)	<.001
Chronic kidney disease	2584 (27.6)	34 529 (20.5)	<.001
Hyperlipidemia	6007 (64.1)	111 923 (66.6)	<.001
Posttraumatic stress disorder	1285 (13.7)	26 220 (15.6)	<.001
Major depressive disorder	2237 (23.9)	44 618 (26.6)	<.001
HIV	66 (0.7)	683 (0.4)	<.001
Hepatitis B or C	377 (4.0)	3353 (2.0)	<.001
Head injury	256 (2.7)	6027 (3.6)	<.001
Problem drinking using AUDIT-C	358 (4.1)	4690 (3.0)	<.001
Tobacco use disorder	827 (8.8)	15 884 (9.5)	.04
Cerebrovascular disease or stroke	2142 (22.9)	36 923 (22.0)	.04
Peripheral vascular disease	1963 (20.9)	29 094 (17.3)	<.001
Congestive heart failure	3278 (35.0)	42 119 (25.1)	<.001
Sleep apnea	1221 (13.0)	24 688 (14.7)	<.001
Rural residence^a^	2601 (28.4)	53 387 (32.4)	<.001
Homelessness	585 (6.2)	10 491 (6.2)	>.99
Region			
Midwest	1779 (19.0)	37 975 (22.6)	<.001
Continental	1533 (16.4)	28 443 (16.9)	
North Atlantic	2156 (23.0)	37 809 (22.6)	
Southeast	2392 (25.5)	39 236 (23.3)	
Pacific	1513 (16.1)	24 586 (14.6)	

Abbreviations: AUDIT-C, Alcohol Use Disorders Identification Test; BMI, body mass index (calculated as weight in kilograms divided by height in meters squared); FIB-4, Fibrosis-4; NA, not applicable.

^a^ Contained missing values. Only complete cases considered.

**Table 2.** Characteristics of Patients With FIB-4 Level Greater Than 2.67 vs Patients With FIB-4 Level 2.67 or Less

Variable	**No. (%)**		*P* value
	FIB-4 > 2.67	FIB-4 ≤ 2.67	
Patients	18 390 (10.4)	159 032 (89.6)	
Age, mean (SD), y	81.04 (9.49)	78.04 (11.09)	<.001
Sex			
Male	18 085 (98.3)	154 274 (97.0)	<.001
Female	305 (1.7)	4758 (3.0)	
Race (White)^a^	13 401 (76.3)	122 591 (80.6)	<.001
Ethnicity (Hispanic or Latino)^a^	1278 (7.2)	10 248 (6.7)	.007
BMI, mean (SD)	25.87 (5.34)	26.78 (5.40)	<.001
Charlson Comorbidity Index, median (IQR)	3.00 (2.00-5.00)	3.00 (2.00-5.00)	<.001
Diabetes	6605 (35.9)	62 162 (39.1)	<.001
Hypertension	14 519 (79.0)	121 981 (76.7)	<.001
Chronic kidney disease	4796 (26.1)	32 317 (20.3)	<.001
Hyperlipidemia	11 915 (64.8)	106 015 (66.6)	<.001
Posttraumatic stress disorder	2487 (13.5)	25 018 (15.7)	<.001
Major depressive disorder	4350 (23.7)	42 505 (26.7)	<.001
HIV	118 (0.6)	631 (0.4)	<.001
Hepatitis B or C	644 (3.5)	3086 (1.9)	<.001
Head injury	475 (2.6)	5808 (3.7)	<.001
Problem drinking using AUDIT-C	620 (3.7)	4428 (3.0)	<.001
Tobacco use disorder	1520 (8.3)	15191 (9.6)	<.001
Cerebrovascular disease or stroke	4157 (22.6)	34 908 (22.0)	.04
Peripheral vascular disease	3721 (20.2)	27 336 (17.2)	<.001
Congestive heart failure	6244 (34.0)	39 153 (24.6)	<.001
Sleep apnea	2377 (12.9)	23 532 (14.8)	<.001
Rural residence^b^	5210 (29.0)	50 778 (32.5)	<.001
Homelessness	1093 (5.9)	9983 (6.3)	.08
Region			
Midwest	3395 (18.5)	36 359 (22.9)	<.001
Continental	3079 (16.7)	26 897 (16.9)	
North Atlantic	4264 (23.2)	35 701 (22.4)	
Southeast	4719 (25.7)	36 909 (23.2)	
Pacific	2933 (15.9)	23 166 (14.6)	

Abbreviations: AUDIT-C, Alcohol Use Disorders Identification Test; BMI, body mass index (calculated as weight in kilograms divided by height in meters squared); FIB-4, Fibrosis-4; NA, not applicable.

^a^ Race and ethnicity categories reported as in the Corporate Data Warehouse.

^b^ Contained missing values. Only complete cases considered.

### Multivariable Analysis: Risk Factors for High FIB-4 Scores 

In the FIB-4 greater than 3.25 model, all variables remained significant except for head injury, major depression, PTSD, ethnicity, HIV, peripheral vascular disease, and sleep apnea (Table 3). Among the categorical variables, history of hepatitis B and C was the risk factor with the highest odds for a high FIB-4 score (OR, 1.79; 95% CI, 1.66-1.91), score followed by AUDIT-C (OR, 1.56; 95% CI, 1.44-1.68), congestive heart failure (OR, 1.48; 95% CI, 1.43-1.54), male gender (OR, 1.43; 95% CI, 1.26-1.61), and chronic kidney disease (OR, 1.11; 95% CI, 1.04-1.17). Diabetes was the categorical variable with the lowest odds (OR, 0.78; 95% CI, 0.73-0.84) with a high FIB-4 score, followed by tobacco use disorder (OR, 0.78; 95% CI, 0.70-0.87), White race (OR, 0.79; 95% CI, 0.73-0.85), living in the Midwestern vs the North Atlantic region (OR, 0.84; 95% CI, 0.77-0.91), hyperlipidemia (OR, 0.84; 95% CI, 0.79-0.89), stroke (OR, 0.85; 95% CI, 0.79-0.91), and rural residence (OR, 0.92; 95% CI, 0.87-0.97). Among continuous variables, an FIB-4 score greater than 3.25; for a 1-unit increase in age and in CCI, the odds of a high FIB-4 score both independently increased by 1.07, while a 1-unit increase in body mass index decreased the odds of FIB-4 greater than 3.25 by 0.97. Using a high FIB-4 cutoff score of 2.67, findings were similar for the variables. When using this cutoff value, hypertension was no longer a significant risk factor within the multivariable model, and peripheral vascular disease and PTSD became significant.

**Table 3.** Logistic Regression for the Overall Cohort With Dementia

Variable	FIB-4 score >3.25		FIB-4 score >2.67	
	OR (95% CI)	*P* value	OR (95% CI)	*P* value
Age (capped at 65 y)	1.07 (1.06-1.09)	<.001	1.09 (1.08-1.10)	<.001
Race, White (vs all others)	0.79 (0.73-0.85)	<.001	0.80 (0.76-0.85)	*<*.001
Ethnicity – Hispanic (vs all others)	1.08 (0.99-1.17)	.12	1.04 (0.97-1.11)	.28
Rural residence (vs urban)	0.92 (0.87-0.97)	.002	0.93 (0.90-0.97)	.001
Gender, male	1.43 (1.26-1.61)	*<*.001	1.51 (1.38-1.64)	*<*.001
Body mass index	0.97 (0.97-0.98)	*<*.001	0.98 (0.97-0.98)	*<*.001
Co-morbidities				
Charlson Comorbidity Index	1.07 (1.06-1.09)	*<*.001	1.04 (1.03-1.05)	*<*.001
Chronic kidney disease	1.11 (1.04-1.17)	.002	1.12 (1.07-1.17)	*<*.001
Hypertension	1.10 (1.04-1.17)	.003	1.03 (0.98-1.08)	.22
Diabetes	0.78 (0.73-0.84)	*<*.001	0.77 (0.73-0.81)	*<*.001
Hyperlipidemia	0.84 (0.79-0.89)	*<*.001	0.89 (0.85-0.93)	*<*.001
Stroke	0.85 (0.79-0.91)	*<*.001	0.88 (0.83-0.92)	*<*.001
Peripheral vascular disease	1.04 (0.98-1.10)	.19	1.07 (1.07-1.12)	.004
Sleep apnea	0.99 (0.92-1.06)	.83	0.98 (0.92-1.03)	.42
Head injury	0.95 (0.80-1.09)	.44	0.92 (0.81-1.03)	.13
Congestive heart failure	1.48 (1.43-1.54)	*<*.001	1.52 (1.48-1.56)	*<*.001
Hepatitis B or C	1.79 (1.66-1.91)	*<*.001	1.79 (1.69-1.89)	*<*.001
Problem drinking, AUDIT-C	1.56 (1.44-1.68)	*<*.001	1.41 (1.32-1.50)	*<*.001
Tobacco use disorder	0.78 (0.70-0.87)	*<*.001	0.77 (0.71-0.84)	*<*.001
HIV	0.87 (0.56-1.18)	.39	1.11 (0.87-1.35)	.39
Major depressive disorder	0.96 (0.91-1.02)	.20	0.96 (0.92-1.00)	.06
Posttraumatic stress disorder	0.93 (0.86-1.00)	.05	0.92 (0.86-0.97)	.001
Regions (vs Northeast)				
Continental	0.99 (0.92-1.07)	.81	1.00 (0.95-1.06)	.92
Midwest	0.84 (0.77-0.91)	*<*.001	0.79 (0.74-0.84)	*<*.001
Pacific	1.07 (0.99-1.15)	.09	1.04 (1.99-1.10)	.14
Southeast	1.07 (1.00-1.14)	.05	1.09 (1.04-1.14)	.001

Abbreviations: AUDIT-C, Alcohol Use Disorders Identification Test; FIB-4, Fibrosis-4.

#### Medical Records Review of All Richmond VAMC Patients From 2009 to 2019

Eighty-nine patients of the cohort were from the Richmond VAMC. The mean (SD) FIB-4 score was 5.12 (2.41), with a platelet count of 124.3 (54.5) × 10^3^/μL; ALT level, 33.0 (24.6) U/L; AST level, 61.5 (41.9) U/L; and true age, 81.2 (10.0) years. Obesity was found in 20 patients (22.4%), diabetes in 27 patients (30.3%), and AUD in 13 patients (14.6%). Hepatitis C status was checked in only 59 patients (66.3%); of these, a positive hepatitis C virus antibody was found in 2 patients (3.3%); 4 of 42 patients (9.5%) with hepatitis B testing were positive.

Thrombocytopenia was found in 76 patients (85.4%) (mean [SD] platelet count, 117.4 [71.3]), 2 of whom had myelodysplastic syndrome. In the remaining 74 patients, an AST to ALT ratio greater than 1 was also seen in 57 patients (64.0%), and hypoalbuminemia was observed in 24 patients (26.9%), suggesting potential advanced liver disease. Liver stiffness was identified in 5 additional patients (3 with ultrasonography and 2 with magnetic resonance imaging), which showed greater than F3 stiffness in all. Imaging showed cirrhosis in 10 patients (5 with nodular liver, 4 with ascites, and 1 with varices). Three additional patients had potential HE but were not treated for it and none underwent an esophagogastroduodenoscopy. Two patients without thrombocytopenia had a liver biopsy that showed no or mild fibrosis. Of the remaining 17 patients with a high FIB-4 score, medical records review no evidence to rule out cirrhosis; all had potential cirrhosis risk factors: 8 patients with AUD, obesity, and type 2 diabetes; 7 with obesity and diabetes; and 2 with AUD only.

Ultimately, we concluded that the definite reasons other than cirrhosis in patients with high FIB-4 scores were found in only 4 patients (2 with liver biopsy and 2 with myelodysplastic syndrome: 5%). The remaining patients (95%) either had evidence of cirrhosis, had risk factors, and/or had no other explanation for their high FIB-4 score.

#### Review of Patients With Dementia in Richmond VAMC

Between January 1 and June 30, 2023, 80 veterans were seen in the geriatric clinics for evaluation of dementia. Using the age-adjusted FIB-4 score (for patients aged >65 years, age was entered as 65 years to calculate the score), 9 patients (11.2%) had an FIB-4 score greater than 2.67 with a mean (SD) of 3.78 (0.67). These patients had a mean platelet count of 129.9 (47.8) × 10^3^/μL; ALT level, 26.7 (13.2) U/L; AST level, 33.3 (11.8) U/L; and age, 79.0 (7.7) years. A local validation cohort of patients with dementia showed a similar percentage of high FIB-4 scores (4.4%-11.2%). Of these 9 patients, 4 had diabetes, 3 had prior AUD, none were obese, 5 were checked for hepatitis C virus antibodies (1 was positive), and 1 already had been diagnosed with cirrhosis and was monitored by the hepatology department. The remaining 8 patients were further evaluated; 1 more had an alternative explanation (myelodysplastic syndrome) for thrombocytopenia. Potential cirrhosis therefore was possibly present in 7 patients (8.7%) (2 patients only had 1 visit with the high FIB-4 score, 5 had high FIB-4 scores on earlier visits along with thrombocytopenia. The geriatricians were encouraged to seek gastroenterology/hepatology investigation.

## Discussion

In a national cohort study of veterans diagnosed with dementia who were not already diagnosed with cirrhosis, we found high FIB-4 scores in 10.3% of patients. The prevalence of potentially undiagnosed cirrhosis, represented by a high FIB-4 score, was higher in non-White and Hispanic vs White patients, urban vs rural veterans, patients with more comorbidities, and patients with AUD and viral hepatitis. The combination of high FIB-4 scores and other risk factors for liver disease in patients with dementia raises the possibility that reversible HE could be a factor associated with cognitive impairment. These findings highlight the potential to enhance cognitive function and quality of life by increasing awareness of risk factors and diagnostic indicators of advanced liver disease that may be associated with HE as a factor or as a differential diagnosis of dementia among clinicians other than liver specialists.

Cirrhosis, HE, and dementia are prevalent in the rapidly increasing older veteran population, creating the potential for overlap and diagnostic confusion.^9,10^ In older patients with risk factors for both cirrhosis and dementia, the lack of clinically applicable biomarkers and diagnostic tests complicates accurate diagnoses and treatment.^10,14^ Therefore, to diagnose or exclude HE as a potential factor associated with cognitive impairment, a strong suspicion for underlying cirrhosis is needed. There is a growing awareness of cirrhosis among primary care physicians, where FIB-4 is being promoted as a tool for screening.^15,16^ The validity of these tools has been established in earlier studies, but a higher prevalence of traditional liver-related risk factors, such as AUD and hepatitis B and C, in the high FIB-4 score groups, and the relative similarity of comparison of patient characteristics between the 2 FIB-4 score thresholds increases confidence in the results. While AUD and viral hepatitis are well known to be associated with advanced liver disease,^17^ these findings should encourage health care professionals taking care of patients with dementia, including primary care, urgent care clinicians, geriatricians, and neurologists, to seek these out and diagnose cirrhosis as a potential factor associated with cognitive dysfunction.

The factors associated with high FIB-4 scores in this population differed in important ways from previous studies of the general population. While some traditional risk factors, such as AUD and viral hepatitis, were associated with high FIB-4 scores, others (eg, diabetes and hyperlipidemia) were not. Although the reasons are unclear, diabetes and hyperlipidemia are associated with vascular dementia, independent of liver disease.^18,19,20^ Depression and PTSD, which are known to be occur more often among people with cirrhosis than the general population, were not associated with high FIB-4 scores in this analysis. This apparent lack of association may have been due to controlling for AUD, which may mediate the associations between PTSD, depression, and cirrhosis.^21,22,23^ Alternatively, dementia may be so often associated with mental health symptoms that the association between mental health symptoms and potential liver disease was not identified in this enriched cohort. While hepatitis C virus and AUD diagnoses were expectedly associated with high FIB-4 scores in this population, metabolic and other comorbidities that have been associated with cirrhosis in other populations^24^ were not associated with high FIB-4, likely because our population was restricted to older patients with dementia whose baseline burden of these diseases differs from that of the general population. Because the factors associated with high FIB-4 scores in the general population may differ in the context of dementia, a higher index of suspicion may be needed to ensure proper diagnosis of liver disease in this complex population. Additionally, in a large sample size, associations with small or clinically irrelevant absolute differences between the groups may still be statistically significant. However, to our knowledge, this study is among the first of its kind to address these potential risk factors; studies are needed to evaluate these risk factors further.

We used the FIB-4 index as a marker of potential liver disease in this study. The FIB-4 index is a scalable, inexpensive, and useful approach to identify patients with possible advanced liver fibrosis or cirrhosis.^12^ We used several validated methods to exclude previously diagnosed cirrhosis in this population.^9^ However, the large number of people with diagnosed dementia and diagnosed cirrhosis represent another population of interest; these have challenges in comanagement because HE and dementia overlap.^9^ The use of FIB-4 scores necessitated exclusion of patients who did not have liver enzyme levels available, and few were excluded for extremes of laboratory test values to avoid confounding. Despite these exclusions, we found that 5% to 10% of veterans with dementia had potential undiagnosed cirrhosis. We found similar patterns with FIB-scores 4 greater than 2.67 or greater than 3.25 when comorbid conditions, as well as sociodemographic characteristics, were compared between patients with and without low or high FIB-4 scores. While the FIB-4 is certainly not a perfect index, it is widely adopted to exclude the presence of advanced liver disease in the general population due to its ease of calculation and interpretation.^25,26^ The FIB-4 score also adds specificity to the alternative of using AST or ALT levels alone, since patients with even advanced liver disease often have laboratory values within the reference ranges.^27^

A particular challenge when studying FIB-4 scores in patients with dementia is the outsized role of older age, which can falsely increase this score. McPherson et al^28^ proposed using actual age with a cutoff level greater than 2.0 in metabolic dysfunction-associated steatotic liver disease fibrosis; a subsequent publication used this modification for people older than 65 years.^29^ However, the ages of patients included in these studies spanned both sides of 65 years, unlike our study where the mean age was 78.35 years. Thus, if these rules were adapted in our cohort of primarily older veterans, a potential overinflation of the FIB-4 score leading to numerous false-positive results may occur. To combat the potential confounding factor of age, we capped patient age at 65 years in the FIB-4 calculation, even though all patients were included. Combined with our high threshold choices of 2.67 and 3.25 for probable advanced fibrosis, this ensured that scores would be conservative and decreased the risk of false-positive results or biased to the null. The main drawback would be neglecting the actual effect of age in patients older than 65 years. In general, research and validation of the FIB-4 index in an older population is limited, and further studies are needed to address this gap. Regardless, the presence of cirrhosis among patients with high FIB-4 scores in this cohort was largely corroborated by medical records review in 2 validation cohorts.

The disparity in potential undiagnosed cirrhosis in veterans with dementia who lived in urban areas, were of Hispanic ethnicity, and were not White, is an important issue. These racial and geographic disparities echo a prior study.^30^ Dementia disproportionately affects Black and Hispanic veterans and is diagnosed later in the disease course in these populations, which has been attributed to lack of high-quality health care access for minoritized populations.^31,32,33^ A lack of access to health care services could also explain potentially underdiagnosed cirrhosis, which reiterates the need for focus on these subpopulations to accurately diagnose cirrhosis and potentially HE.^34,35,36^

We focused on veterans with dementia with a high FIB-4 score due to the important therapeutic implications of a missed cirrhosis diagnosis and, therefore, a missed HE diagnosis. An earlier granular study showed that patients with combined amnestic (related to dementia) and anamnestic (related to cirrhosis) cognitive dysfunction in cirrhosis have a worse quality of life, which improves after HE therapy.^14^ Moreover, there is a major overlap between HE and dementia in veterans with diagnosed cirrhosis.^9^ Even if the ultimate diagnosis of HE is not considered to be a factor associated with the cognitive dysfunction in those diagnosed with cirrhosis, hepatocellular cancer and other cirrhosis complications also need to be treated or monitored.^37^

### Strengths and Limitations

The advantages of the CDW are the national scope, potential to study geographic variations, and the diverse population of veterans whose primary source of health care is the VHA system. The limitations are the use of coding and relative lack of granularity in the cirrhosis and dementia diagnoses. Also, the relative proportion of HE in those with undiagnosed cirrhosis, and the potential HE treatment needs to be addressed, which can only be done by an individual patient assessment. Therefore, we further analyzed this with a detailed medical records review of earlier (CDW) and current dementia cohorts. This study had the unintended positive consequence of increasing the awareness of collaborating geriatricians, who were advised to refer the patients to hepatology services. However, more work is needed to increase recognition of cirrhosis and optimize care for people with a known overlap between cirrhosis and dementia.

## Conclusions

The findings of this cohort study suggest that, in a national veterans cohort of patients with dementia, 5% to 10% of the patients have laboratory values suggestive of possible undiagnosed cirrhosis that could implicate HE as a contributor to overall cognitive impairment. Patients in this study with possibly undiagnosed cirrhosis were more likely to be of races other than White, Hispanic, and urban-dwelling, and more likely to have AUD and viral hepatitis. These percentages of affected patients were corroborated with medical records review of 2 separate validation cohorts. In conclusion, FIB-4 should be considered as a screening tool to detect cirrhosis and potential HE in older veterans with dementia. Those with high scores (eg, >2.67) should be considered for further evaluation and treatment.

## Notes

Supplement 1. **eAppendix.** *ICD* Codes Used

Supplement 2.
Data Sharing Statement
