# Supplementary material for: Undiagnosed Cirrhosis and Hepatic Encephalopathy in a National Cohort of Veterans With Dementia
Source: JAMA Netw Open. 2024 Jan 31;7(1):e2353965. doi: 10.1001/jamanetworkopen.2023.53965 (PMC10831576; doi:10.1001/jamanetworkopen.2023.53965)
Supplement: Supplement 1. — eAppendix. ICD Codes Used [file jamanetwopen-e2353965-s001.pdf]

Supplementary Online Content

Bajaj JS, Silvey SS, Rogal S, et al. Undiagnosed cirrhosis and hepatic encephalopathy in a national cohort of veterans with dementia. *JAMA Netw Open*. 2024;7(1):e2353965. doi:10.1001/jamanetworkopen.2023.53965

eAppendix. *ICD* Codes Used

This supplementary material has been provided by the authors to give readers additional information about their work.

© 2024 Bajaj JS et al. *JAMA Network Open.*

eAppendix. *ICD* codes used

Cirrhosis ICD Codes :

ICD-10 :

I85.00, I85.01, I85.10, I85.11, K65.2, K70.11, K70.30, K70.31, K70.40, K70.41, K71.51, K71.7, K72.10, K72.11, K74.4, K74.60. K74.69, K76.6, K76.7, K76.81

ICD-9 :

456.0, 456.1, 456.20, 456.21, 567.23, 571.2, 571.5, 572.2, 572.3, 572.4

Dementia ICD Codes :

ICD-10 :

G30.1, G31.01, G31.09, R84.81, G30.9, G30.8, F03.90, F02.C4, F02.C3, F02.C2, F02.C18, F02.C11, F02.C0, F02.B4, F02.B3, F02.B2, F02.B18, F02.B11, F02.B0, F02.A4, F02.A3, F02.A2, F02.A18, F02.A11, F02.A0, F02.84, F02.83, F02.82, F02.818, F02.811, F02.81, F02.80, F01.C4, F01.C3, F01.C2, F01.C18, F01.C11, F01.C0, F01.B4, F01.B4, F01.B3, F01.B2, F01.B18, F01.B11, F01.B0, F01.A4, F01.A3, F01.A2, F01.A18, F01.A11, F01.A0, F01.54, F01.53, F01.52, F01.518, F01.511, F01.51, F01.50, F04., F05., F06.0, F06.1, F06.8, G13.2, G13.8, G31.1, G31.2, G91.4, G94.

ICD-9 :

331, 331.1, 331.11, 331.19, 331.2, 331.7, 290, 290.1, 290.11, 290.12, 290.13, 290.2, 290.21, 290.3, 290.4, 290.41, 290.42, 290.43, 290.8, 290.9, 294, 294.1, 294.11, 294.8

Alcohol Use Disorder Codes :

ICD-10 :

F10.10, F10.120, F10.121, F10.129, F10.14, F10.150, F10.151, F10.159, F10.180, F10.181, F10.182, F10.188, F10.19, F10.20, F10.220, F10.221, F10.229, F10.230, F10.231, F10.232, F10.239, F10.24, F10.250, F10.251, F10.259, F10.26, F10.27, F10.280, F10.281, F10.282, F10.288, F10.29, F10.920, F10.921, F10.929, F10.94, F10.950, F10.951, F10.959, F10.96, F10.97, F10.980, F10.981, F10.82, F10.988, F10.99

ICD-9 :

303.01, 303.02, 303.9, 303.91, 303.92, 305, 305.01, 305.02

© 2024 Bajaj JS et al. *JAMA Network Open.*
