# Supplementary material for: Undiagnosed Cirrhosis and Hepatic Encephalopathy in a National Cohort of Veterans With Dementia
Source: JAMA Netw Open. 2024 Jan 31;7(1):e2353965. doi: 10.1001/jamanetworkopen.2023.53965 (PMC10831576; doi:10.1001/jamanetworkopen.2023.53965)
Supplement: Supplement 2. — Data Sharing Statement [file jamanetwopen-e2353965-s002.pdf]

Data Sharing Statement

Bajaj. Undiagnosed Cirrhosis and Hepatic Encephalopathy in a National Cohort of Veterans With Dementia. *JAMA Netw Open*. Published January 31, 2024.
doi:10.1001/jamanetworkopen.2023.53965

Data
Data available: No
